# An Externally-Validated Dynamic Nomogram Based on Clinicopathological Characteristics for Evaluating the Risk of Lymph Node Metastasis in Small-Size Non-small Cell Lung Cancer

**DOI:** 10.3389/fonc.2020.01322

**Published:** 2020-08-07

**Authors:** Yijun Wu, Chang Han, Zhile Wang, Liang Gong, Jianghao Liu, Yuming Chong, Xinyu Liu, Naixin Liang, Shanqing Li

**Affiliations:** ^1^Department of Thoracic Surgery, Peking Union Medical College Hospital, Chinese Academy of Medical Sciences and Peking Union Medical College, Beijing, China; ^2^Peking Union Medical College, Eight-year MD Program, Chinese Academy of Medical Sciences, Beijing, China; ^3^Department of Radiology, Peking Union Medical College Hospital, Chinese Academy of Medical Sciences and Peking Union Medical College, Beijing, China

**Keywords:** non-small cell lung cancer, LASSO regression, dynamic nomogram, lymph node metastasis, external validation

## Abstract

**Background:** Lymph node metastasis (LNM) status is of key importance for the decision-making on treatment and survival prediction. There is no reliable method to precisely evaluate the risk of LNM in NSCLC patients. This study aims to develop and validate a dynamic nomogram to evaluate the risk of LNM in small-size NSCLC.

**Methods:** The NSCLC ≤ 2 cm patients who underwent initial pulmonary surgery were retrospectively reviewed and randomly divided into a training cohort and a validation cohort as a ratio of 7:3. The training cohort was used for the least absolute shrinkage and selection operator (LASSO) regression to select optimal variables. Based on variables selected, the logistic regression models were developed, and were compared by areas under the receiver operating characteristic curve (AUCs) and decision curve analysis (DCA). The optimal model was used to plot a dynamic nomogram for calculating the risk of LNM and was internally and externally well-validated by calibration curves.

**Results:** LNM was observed in 12.0% (83/774) of the training cohort and 10.1% (33/328) of the validation cohort (*P* = 0.743). The optimal model was used to plot a nomogram with six variables incorporated, including tumor size, carcinoembryonic antigen, imaging density, pathological type (adenocarcinoma or non-adenocarcinoma), lymphovascular invasion, and pleural invasion. The nomogram model showed excellent discrimination (AUC = 0.895 vs. 0.931) and great calibration in both the training and validation cohorts. At the threshold probability of 0–0.8, our nomogram adds more net benefits than the treat-none and treat-all lines in the decision curve.

**Conclusions:** This study firstly developed a cost-efficient dynamic nomogram to precisely and expediently evaluate the risk of LNM in small-size NSCLC and would be helpful for clinicians in decision-making.

## Introduction

Lung cancer is the most common cause of cancer-related death worldwide in recent years (1). As computed tomography (CT) becomes the main means of screening for high-risk populations of lung cancer, the detection rate for small-size lung cancer has been increasing (2). The standard treatment for early-stage lung cancer is lobectomy with systematic lymph node dissection (LND) (3), but whether lobectomy with systematic LND is necessary for all these patients remains unclear. The sublobar resection (wedge resection and segmentectomy) has been used for early-stage NSCLC, especially for patients with impaired pulmonary function reserve (4). Moreover, compared with those who underwent systematic LND, patients that received selective LND also presented a lower incidence of perioperative complications and similar survival (5, 6). However, these sublevel surgical procedures would be more likely to cause tumor residual, since occult LNM occurred not rarely, even though in small-size NSCLC (7–11). Of possibly greater concern is the occurrence of micrometastases in histologically negative lymph nodes from early-stage NSCLC patients (10). Thus, developing reliable methods for evaluating the risk of LNM is of great importance, and would be helpful for decision making of medical management.

CT and positron emission tomography/computed tomography (PET/CT) are widely used for noninvasive nodal staging, but these methods have limited accuracy. Invasive node staging approaches, such as mediastinoscopy and endobronchial ultrasound transbronchial needle aspiration (EBUS-TBNA), may not be cost-efficient for small-size NSCLC patients. However, there is still no generally-accepted method for precisely evaluating the risk of LNM in early-stage NSCLC. The clinicopathological characteristics and risk factors associated with LNM remain unclear. We aimed to analyze the clinicopathological characteristics related to LNM and to develop and validate a cost-efficient dynamic nomogram for evaluating the risk of LNM in patients with small-size NSCLC.

## Materials and Methods

### Patient Enrollment

**Figure 1:**
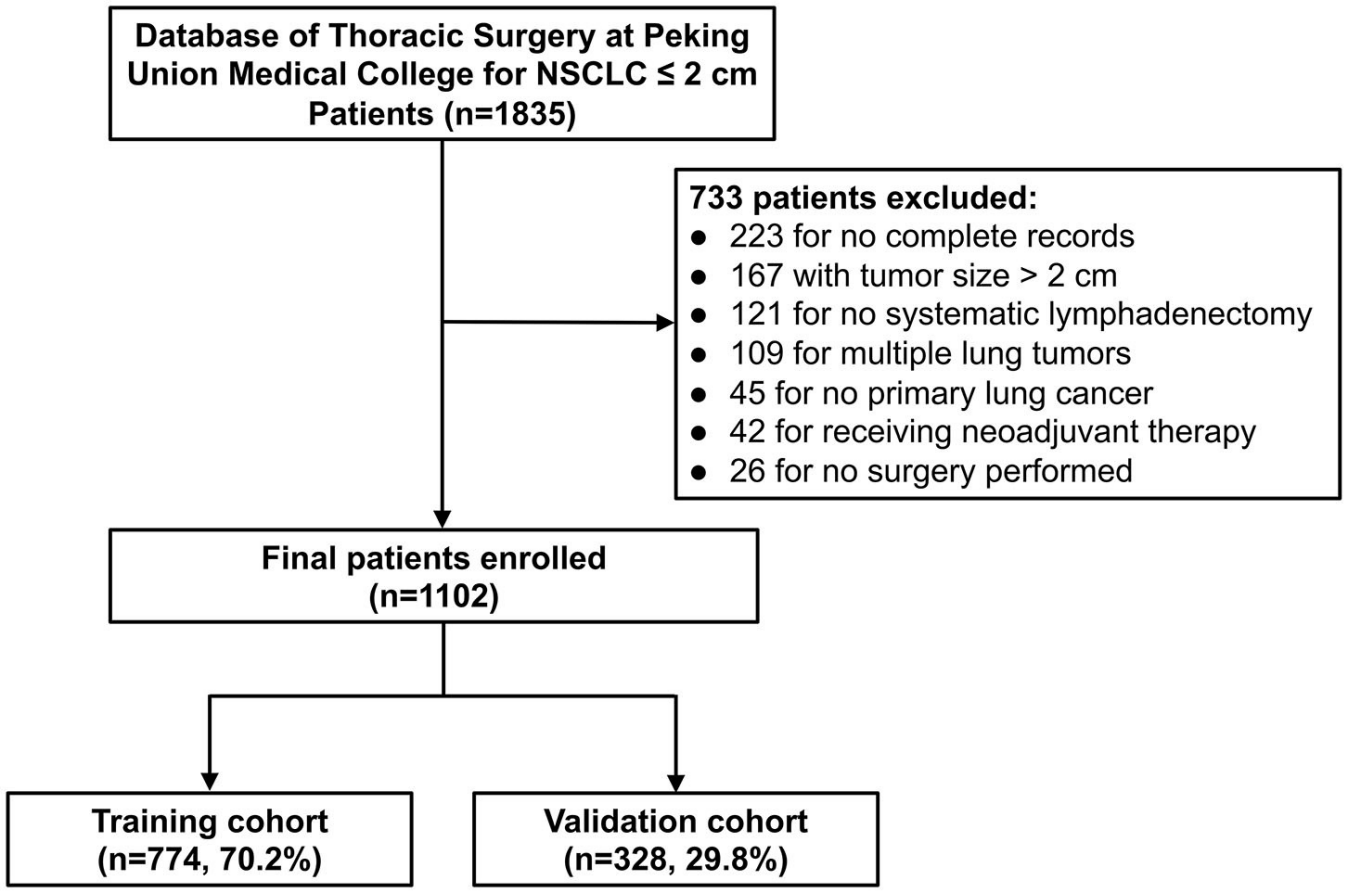
Flowchart of the patients for the training and validation cohorts.

From January 2013 to June 2019, NSCLC patients in Peking Union Medical College Hospital (PUMCH) were retrospectively reviewed (Figure 1). The eligibility criteria were: (1) single cancer lesion; (2) ≤ 2 cm in maximal diameter on CT; (3) receiving lung resection (lobectomy or sublobar resection) with systematic LND; (4) complete pathological information; (5) not receiving neoadjuvant chemotherapy or radiotherapy before surgery. This study was approved by the Ethics Committee of Peking Union Medical College Hospital. All patients signed informed consent forms before operation.

### Clinicopathological Characteristics

All clinical information, including gender, age, smoking status, and serum carcinoembryonic antigen (CEA), was collected before the operation. The CT were performed within 60 days before surgery, and the imaging features involved maximal tumor size, imaging density, and specific signs such as speculation, vessel convergence, lobulation, pleural indentation, and calcification. The CT images were reviewed by two thoracic surgeons and one radiologist independently. The final conclusion was made by consensus reading if disagreement occurred between them. The tumor imaging density was grouped into pure ground glass nodule (pGGN), mixed GGN (mGGN), and solid lesions. mGGN was defined as the presence of a solid component within the nodule at the mediastinal window level of CT. Then, the mGGN was further divided into two groups according to the ratio of the maximal diameter of the solid component to the maximal diameter of the tumor area (the cutoff value was set as 50%). The pathological findings were recorded from paraffin-embedded surgery specimens by pathological experts from PUMCH. Patients' pathological N status was confirmed according to the 8th edition TNM Classification for lung cancer (12). The presence of lymphovascular invasion (LVI) and pleural invasion were also included.

### Surgical Procedure

For patients with a tumor size <8 mm, lung resection was considered for those with high risk of malignancy after follow-up or at the demand of patients. Sublobar resection (wedge resection or segmentectomy) was considered for patients with a peripheral pGGN tumor or those with poor lung function reserve or comorbidities. For others, standard lobectomy with systematic LND was recommended firstly. In addition to N_1_ nodes (#10, #11, #12, #13, and #14), systematic LND included 2R, 4R, 3A, 3P, #7, #8, and #9 for tumors located in the right lung and 4L, #5, #6, #7, #8, and #9 for tumors located in the left lung, if possible.

### Statistical Analysis

Patients were randomly divided into a training cohort and a validation cohort as a ratio of 7:3. Using the training cohort, the least absolute shrinkage and selection operator (LASSO) regression was performed to select optimal predictive variables for LNM. Then the logistic regression models were constructed by incorporating these variables. The models' performance in both the training cohort and validation cohort included discrimination and calibration. Discrimination is the predictive accuracy to distinguish patients with LNM from those without LNM and can be measured by the area under the receiver operating characteristic (ROC) curve (AUC). Calibration curves were plotted using 1,000 bootstrap resamples, which reflected the model's agreement between the predicted probability and the actual probability. Decision curve analysis (DCA) was used to assess the model's clinical usefulness. Based on the logistic regression model, a dynamic nomogram was developed, presenting a specific system for calculating the risk of LNM.

Continuous data were expressed as median with interquartile (IQR). Statistical analysis was performed using IBM SPSS 25.0 (SPSS Inc; Chicago, IL, USA) and R software version 3.6.3. Pearson's Chi square test was used for categorical data analysis (Fisher exact test was used if necessary), while Mann–Whitney *U*-test were used to compare quantitative parameters. *P* < 0.05 were considered statistically significant. All statistical analyses were two-sided.

## Results

### Patient Characteristics

A total of 1,102 patients met the inclusion criteria, including 403 males and 699 females. Among them, 116 patients (10.5%) were node-positive (pN_+_), and 986 (89.5%) had no lymph node metastasis (pN_0_) by final histopathology. The median age was 58 (IQR: 51–65) years, and the median tumor size on CT was 1.3 (IQR: 1.0–1.7) cm. Lobectomy was performed for 870 patients and sublobar resection for 232 patients. The two cohorts' data of demographic characteristics and variables are shown in Table 1. The training cohort consisted of 774 (71.6%) patients, while the validate cohort included 328 (28.4%) patients. The LNM rate was 12.0% (83/774) in the training cohort vs. 10.1% (33/328) in the validation cohort (*P* = 0.743). The two cohorts were similar in most of characteristics except smoking history (*P* = 0.049) and imaging density (*P* = 0.039), the distribution of which were with slightly significant difference.

**Table 1:** Demographic and clinicopathological characteristics in the training and validation cohorts.

**Characteristic**	**Total**	**Training**	**Validation**	***P*-value**
	**(*N* = 1,102)**	**cohort**	**cohort**	
**LNM**				
Positive	116	83 (71.6)	33 (28.4)	0.743
Negative	986	691 (70.1)	295 (29.9)	
**Age, years**				
<58 (median)	525	379 (72.2)	146 (27.8)	0.176
≥58	577	395 (68.5)	182 (31.5)	
**Sex**				
Male	403	294 (73.0)	109 (27.0)	0.134
Female	699	219 (68.7)	480 (31.3)	
**Smoking history**				
Yes	218	165 (75.7)	53 (24.3)	0.049
No	884	609 (68.9)	275 (31.1)	
**Tumor location**				
Left	641	459 (71.6)	182 (28.4)	0.241
Right	461	315 (68.3)	146 (31.7)	
**Imaging density**				
pGGN	431	284 (65.9)	147 (34.1)	0.039
mGGN	476	346 (72.7)	130 (27.3)	
SN	195	144 (73.8)	51 (26.2)	
**Spiculation sign**				
Present	587	415 (70.7)	172 (29.3)	0.72
Absent	515	359 (69.7)	156 (30.3)	
**VCS**				
Present	234	162 (69.2)	72 (30.8)	0.705
Absent	868	612 (70.5)	256 (29.5)	
**Lobulation sign**				
Present	403	297 (73.7)	106 (26.3)	0.056
Absent	699	477 (68.2)	222 (31.8)	
**Pleural indentation**				
Present	294	214 (72.8)	80 (27.2)	0.263
Absent	808	560 (69.3)	248 (30.7)	
**Calcification sign**				
Present	22	17 (77.3)	5 (22.7)	0.466
Absent	1,080	757 (70.1)	323 (29.9)	
**Histological type**				
ADC	1056	739 (70.0)	317 (30.0)	0.375
Non-ADC	46	35 (76.1)	11 (23.9)	
**LVI**				
Present	31	24 (70.0)	7 (30.0)	0.375
Absent	1,071	750 (77.4)	321 (22.6)	
**Pleural invasion**				
Present	109	78 (71.6)	31 (28.4)	0.75
Absent	993	696 (70.1)	297 (29.9)	
**Surgical procedure**				
Sublobar resection	232	162 (69.8)	70 (30.2)	0.878
Lobectomy	870	612 (70.3)	258 (29.7)	
Tumor size	1.3 [1.0–1.7]	1.3 [1.0–1.7]	1.3 [1.0–1.6]	0.344
CEA, ng/ml	1.89 [1.33–2.55]	1.89 [1.30–2.61]	1.89 [1.36–2.47]	0.639

*LNM, lymph node metastasis; pGGN, pure ground glass nodule; mGGN, mixed GGN; SN, solid nodule; VCS, vessel convergence sign; ADC, adenocarcinoma; LVI, lymphovascular invasion; CEA, carcinoembryonic antigen*.

### Variable Selection for Constructing Models

**Figure 2:**
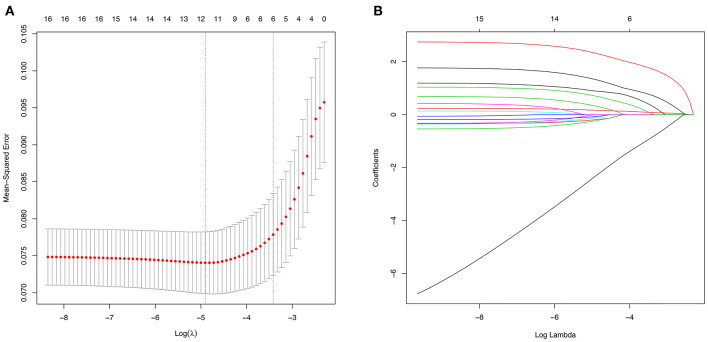
Variable selection using the least absolute shrinkage and selection operator (LASSO) regression model. **(A)** The selection of optimal predictive variables by 5-fold cross-validation. The left and right dotted vertical lines represent the optimal values of lambda when using the minimum criterion and the one-fold standard error of minimum criterion, respectively. **(B)** LASSO coefficients of the 15 variables.

Using LASSO regression in the training cohort, 15 variables in Table 1 were reduced to 6 when using 1 standard error (1–SE) criterion, and reduced to 11 when using the minimum error criterion (Figure 2). Then two logistic regression models, which were marked as Model1 and Model2, were developed by incorporating the 6 variables and 11 variables in the training cohort, respectively. The details for selected variables are shown in Table 2, including tumor size (OR: 3.889, 95%CI: 1.878–8.052, *P* < 0.001), CEA (OR: 1.247, 95%CI: 1.110–1.402, *P* < 0.001), imaging density, histology (OR: 3.583, 95%CI: 1.466–8.757, *P* = 0.005), lymphovascular invasion (OR: 11.979, 95%CI: 4.479–32.039, *P* < 0.001), and pleural invasion (OR: 2.406, 95%CI: 1.223–4.731, *P* < 0.001).

**Table 2:** The logistic regression models using the selected optimal predictive variables in the training cohort.

	**Variable**	**Coefficient**	**OR (95%CI)**	***P*-value**
Model1	Size, cm	1.358	3.889 (1.878–8.052)	<0.001
	CEA, ng/ml	0.221	1.247 (1.110–1.402)	<0.001
	Imaging density (Ref: SN)			
	pGGN	−18.433	<0.001	0.994
	mGGN	0.362	1.436 (0.772–2.669)	0.253
	Non-ADC (Ref: ADC)	1.276	3.583 (1.466–8.757)	0.005
	LVI	2.483	11.979 (4.479–32.039)	<0.001
	Pleural invasion	0.878	2.406 (1.223–4.731)	0.011
Model2	Size, cm	1.732	5.655 (2.623–12.190)	<0.001
	CEA, ng/ml	0.234	1.263 (1.119–1.426)	<0.001
	Imaging density (Ref: SN)			0.588
	pGGN	−18.622	<0.001	0.994
	mGGN	0.335	1.398 (0.739–2.644)	0.303
	Non-ADC (Ref: ADC)	1.153	3.169 (1.243–8.079)	0.016
	LVI	2.789	16.26 (5.654–46.763)	<0.001
	Pleural invasion	1.043	2.837 (1.385–5.810)	0.004
	Age ≥ 58, years (Ref: <58)	0.683	1.981 (1.108–3.541)	0.021
	Spiculation sign	−0.351	0.704 (0.355–1.393)	0.313
	VCS	−0.545	0.580 (0.259–1.300)	0.186
	Lobulation sign	−0.207	0.813 (0.425–1.554)	0.531
	Pleural indentation	−0.350	0.705 (0.365–1.361)	0.298

*CEA, carcinoembryonic antigen; SN, solid nodule; pGGN, pure ground glass nodule; mGGN, mixed GGN; ADC, adenocarcinoma; LVI, lymphovascular invasion; VCS, vessel convergence sign. Ref, reference*.

### Model Validation

**Figure 3:**
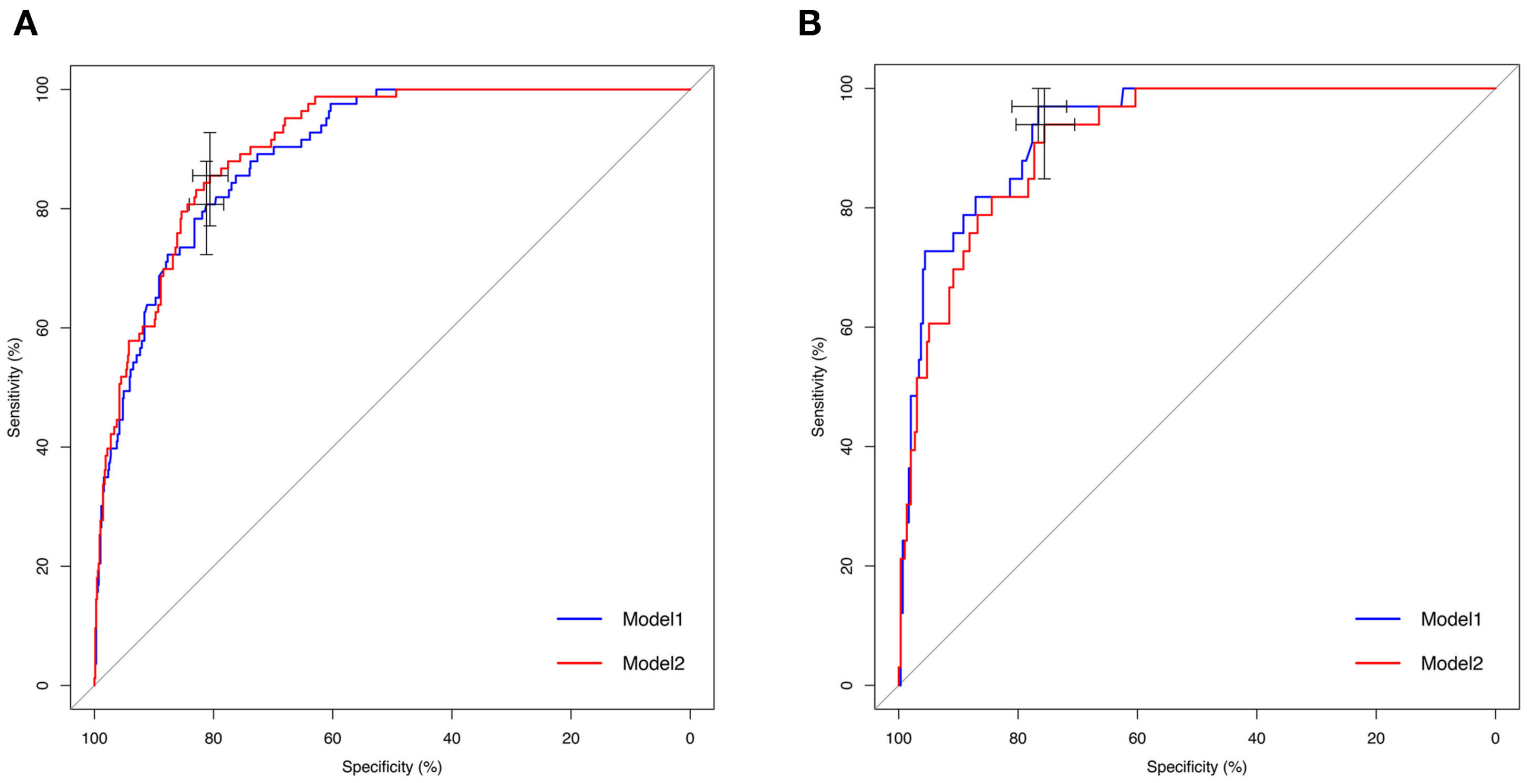
Receiver operating characteristic (ROC) curves of Model1 and Model2 for predicting lymph node metastasis in the training and validation cohorts. **(A)** ROC curve in the training cohort. Area under ROC curve (AUC): 0.895 (95%CI: 0.866–0.925; sensitivity: 80.7%, specificity: 81.2%) in Model 1 vs. 0.907 (95%CI: 0.880–0.934; sensitivity: 85.5%, specificity: 80.6%) in Model2 (*P* = 0.171). **(B)** ROC curve in the validation cohort. AUC: 0.931 (95%CI: 0.896–0.967; sensitivity: 97.0%, specificity: 76.6%) in Model1 vs. 0.915 (95%CI: 0.875–0.955; sensitivity: 93.9%, specificity: 75.6%) in Model2 (*P* = 0.047).

**Figure 4:**
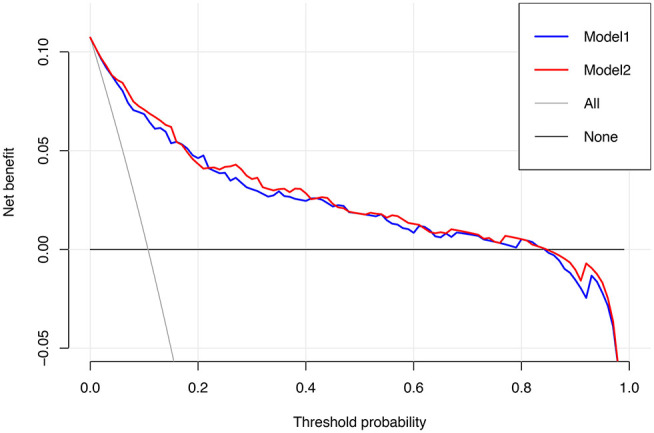
Decision curve for Model1 and Model2. The treat-all line represents the net benefits of giving all patients lobectomy with systematic lymph node dissection, and the treat-none line represents the net benefits of giving no patient the above-mentioned surgical procedure.

**Figure 5:**
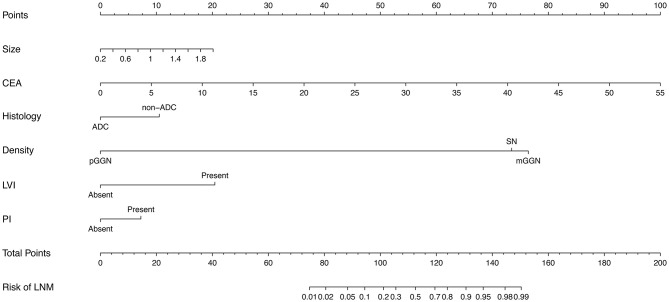
The nomogram for predicting the risk of lymph node metastasis in patients with NSCLC ≤ 2 cm, based on six clinicopathological variables (tumor size, CEA, histological type, imaging density, LVI, PI). CEA, carcinoembryonic antigen; ADC, adenocarcinoma; pGGN, pure ground glass nodule; mGGN, mixed GGN; SN, solid nodule; LVI, lymphovascular invasion; PI, pleural invasion.

**Figure 6:**
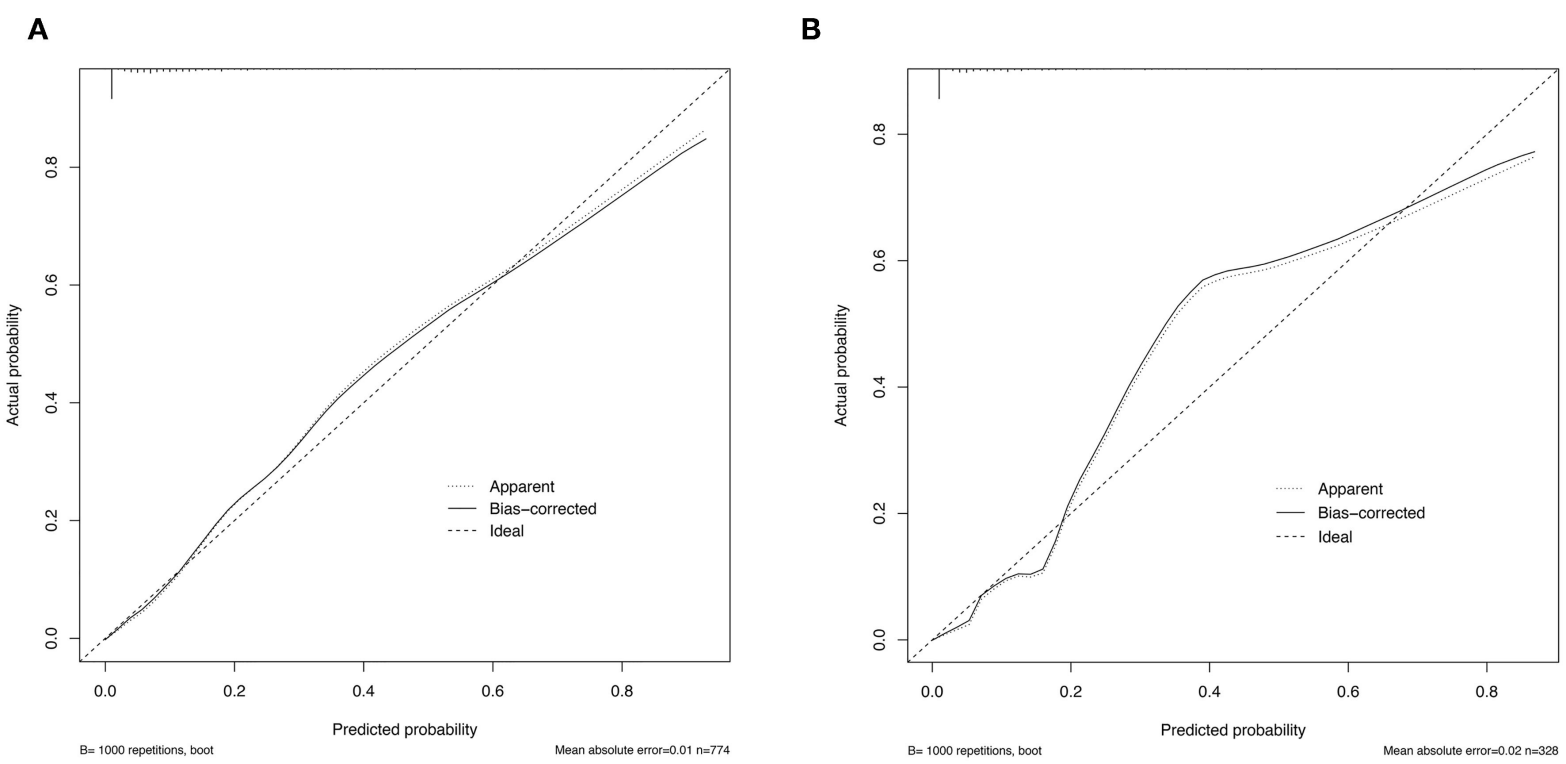
Calibration curves for the nomogram model in the training cohort **(A)** and validation cohort **(B)**.

To assess the predictive ability of models, the ROC curves in both the training cohort and validation cohort were plotted (Figure 3). In the training cohort, the AUC for Model1 was 0.895 (95%CI: 0.866–0.925), similar to 0.907 (95%CI: 0.880–0.934) for Model2 (*P* = 0.171; Figure 3A). However, Model1 (AUC = 0.931, 95%CI: 0.896–0.967) performed significantly better than Model2 (AUC = 0.915, 95%CI: 0.875–0.955) in the validation cohort (*P* = 0.047; Figure 3B). In addition, decision curve showed that two models presented similar net benefits at the entire range of threshold probabilities, with the better performance than the two extreme lines (treat-none and treat-all) when the threshold probability was 0–0.8 (Figure 4). Thus, with fewer variables incorporated, Model1 was selected as a better model and used to develop a nomogram for calculating the risk of LNM (Figure 5). Then calibration curves were plotted for internal and external validation, showing the great calibration of the nomogram model in both training and validation cohorts (Figure 6). Additionally, a dynamic nomogram application (https://nomogramwyj.shinyapps.io/27978155ff984a9f9616812a465b802c) was also developed, which can be conveniently available to clinicians and patients worldwide. Using the application, one patient's risk probability plus 95%CI of LNM can be obtained immediately when imputing his information of the six variables we identified (shown in Supplement Figure 1). The R code and data for the application were attached in Supplement Data Sheet 1.

**Figure 7:**
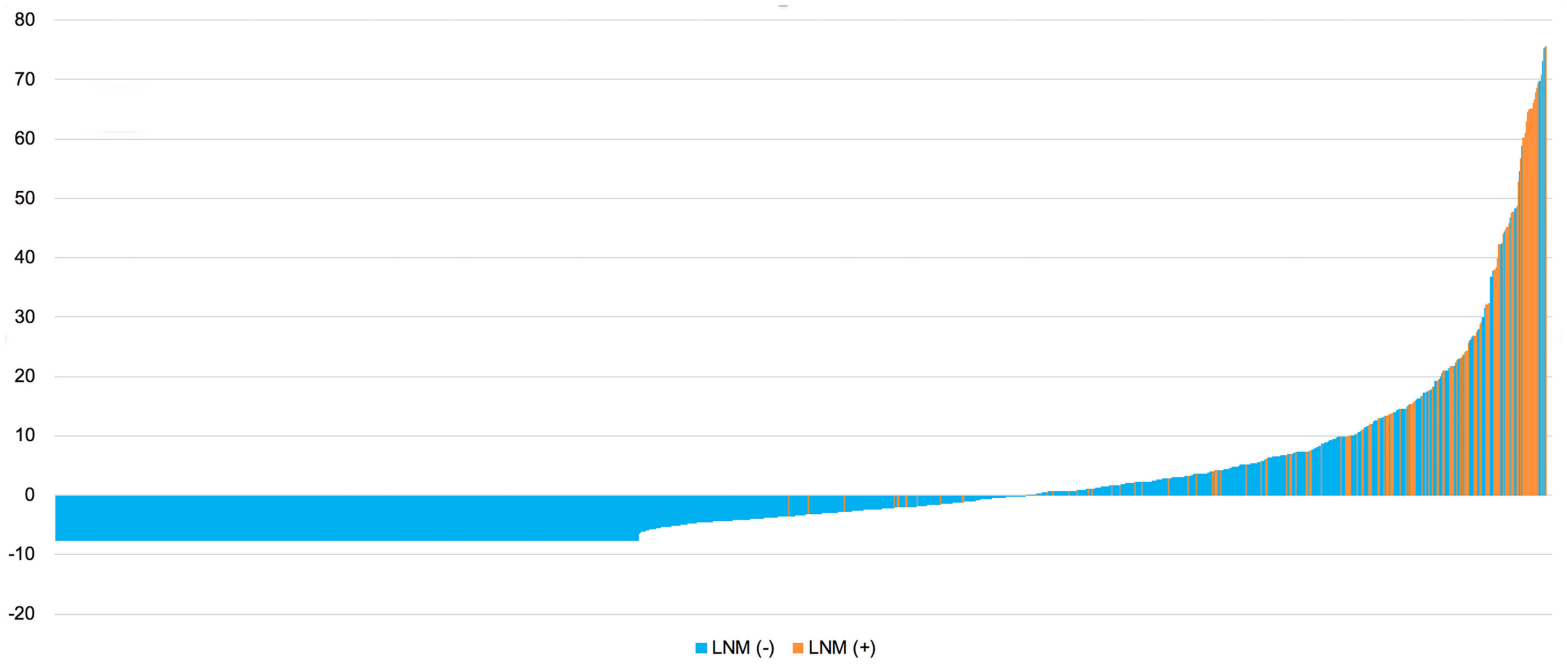
Standardized predictive risk probabilities of LNM calculated by the nomogram for all patients. LNM, lymph node metastasis.

Based on the nomogram model, each patient's risk probability of LNM was calculated. The optimal cutoff point to distinguish between LNM (–) and LNM (+) was 0.092. All patients' predictive risk probabilities from the nomogram was standardized using the following formula: (risk probability−0.092)/standard deviation. Then the standardized risk of each patient was shown in Figure 7. The *x*-axis represents each patient, while *y*-axis represents the standardized risk probabilities from the nomogram.

## Discussion

In this study, single NSCLC ≤ 2 cm were retrospectively reviewed for the development and validation of a dynamic nomogram for evaluating the risk of LNM in small-size NSCLC. To our knowledge, this study was the first one to develop a dynamic nomogram for the evaluation of LNM in lung cancer. For patients whose tumors were staged as T_1_N_0_M_0_, the standard therapy has been considered as lobectomy with systematic LND (3). In recent years, with the rapid development and employment of radiographical screening methods, increasing numbers of small-size lung carcinomas have been discovered. Several studies have reported no significant difference in survival between patients with stage I NSCLC who underwent sublobar resection (wedge resection and segmentectomy) and standard lobectomy, especially for NSCLC ≤ 2 cm (4, 13, 14). SABR (15) and RFA (16), which have also become alternative treatments for small-size NSCLC, might be more appropriate for elderly or inoperable patients than surgery. However, compared to standard lobectomy, sublobar resection and other alternatives might lead to a higher risk of recurrence and a worse survival (17); one reason for this difference might be attributed to occult LNM and micrometastasis, which were not rare in small-size NSCLC (7–11). According to studies from Shi et al. (18) and Yu et al. (19) the incidence rates of LNM in NSCLC ≤ 2 cm were up to 14.1 and 10.2%, respectively, which were similar to the result in our study (10.5%, 116/1,102). Therefore, it is necessary to evaluate the risk of LNM in patients with small-size NSCLC. The reliable predictive methods are highly required.

Unlike the conventional univariate analysis, LASSO regression that we used aimed to select variables for logistic regression to avoid overfitting. Then two models were developed with different number of variables. Both of models presented very high AUCs in ROC curves (Figure 3) and similar clinical usefulness in the decision curve (Figure 4). The Model1, with much fewer variables incorporated, was used to plot the nomogram. The nomogram was a novel cost-efficient tool for precisely calculating the risk of LNM (Figure 5). It has a user-friendly interface and is very convenient to apply in helping making clinical decisions. Our nomogram model was also well-calibrated in both internal and external validation (Figure 6), with the mean absolute error of 0.01 and 0.02, respectively. Thus, based on the nomogram, each patient's risk for LNM can be accurately calculated according to the six variables, which were tumor size, CEA level, imaging density, pathological type of NSCLC (adenocarcinoma or non-adenocarcinoma), lymphovascular invasion and pleural invasion.

Previously, our (20) and other studies (19, 21–23) have found that several preoperative factors might be associated with the occurrence of LNM in small-size NSCLC, including serum CEA, tumor size and imaging features such as imaging density. Tumor size was identified as an important predictor for LNM. The incidence rate of LNM increased as tumor size increased. In our nomogram model, the larger tumor size (OR: 3.889, 95%CI: 1.878–8.052, *P* < 0.001) was an independent risk factor for LNM. Okada et al. (24) thought of tumor size as a predictor for sublobar resection, but in our study, only those patients with tumors ≤ 0.5 cm were not found to have positive nodes. It is not advisable to choose surgical procedure and postoperative management solely by tumor size. Other factors should be considered. Similar to previous studies (19, 22), a higher serum CEA (OR: 1.247, 95%CI: 1.110–1.402, *P* < 0.001) was also associated with a higher LNM rate in our study. Thus, the serum CEA level might be helpful for LNM prediction and should be listed as a routine test for NSCLC patients. In addition, tumor imaging density was also considered to be an important risk factor for LNM since none of the patients with a pGGN tumor were found to be node-positive in our study. The solid component on CT has been confirmed as having a more invasive ability for lung tumors and indicated a significantly higher LNM rate than pGGN (21), which was further consolidated by our study. Therefore, pGGN can be a strongly effective predictor for node negativity in small-size NSCLC.

Moreover, this study also indicated that pathology had a strong association with LNM in patients with small-size NSCLC. Non-adenocarcinomas (OR: 3.583, 95%CI: 1.466–8.757, *P* = 0.005) were more likely to have LNM than adenocarcinomas, although there were only limited quantities of their cases. Furthermore, the presence of lymphovascular invasion (OR: 11.979, 95%CI: 4.479–32.039, *P* < 0.001) and pleural invasion (OR: 2.406, 95%CI: 1.223–4.731, *P* < 0.001) significantly indicated LNM. Therefore, when lymphovascular invasion or pleural invasion was present, even if the histological results showed the dissected lymph nodes were negative, surgeons should be more alert to the occurrence of LNM. If possible, pathologists might observe these characteristics intraoperatively and help surgeons make decisions of surgical procedure, especially for those patients who are difficult to decide between radical and sublevel resection.

Our previous study developed machine learning-based models for the preoperative prediction of LNM (20), but the LNM status should be furtherly evaluated after surgery, with considering histology, especially for patients with negative nodes. The current surgery methods cannot guarantee the removal of all tumor cell-invaded lymph nodes. Additionally, with the increasing application of sublevel resection, the undetected occult LNM and micrometastasis might occur more frequently, likely to cause recurrence and worse survival. Furthermore, the advanced immunotherapy for NSCLC patients may require an accurate N stage, since a discrepant programmed death-ligand 1 (PD-L1) expression was observed between primary tumors and nodal metastases of NSCLC (25). The small-size NSCLC patients with nodal metastases are the potential population that may benefit from adjuvant immunotherapy, and the nomogram we developed can be helpful for identifying optimal candidates for immunotherapy. Thus, based on our nomogram model, the patients with high risk of LNM could be precisely selected for closer postoperative follow-up and timely medical intervention, which might improve prognosis.

In addition, some methodological innovations were indicated in our study. The nomogram was rarely used to predict LNM in lung cancer. Jiang et al. (26) developed a nomogram for predicting occult N2 LNM in squamous cell lung cancer, but the number of cases was very limited and only involved squamous carcinomas. Some previous studies have developed nomogram models using radiomics for predicting LNM (27, 28). However, identifying radiomics features requires special techniques, and the radiomics models are hard to be clinically applied. The dynamic nomogram we developed was based on clinicopathological characteristics that were routinely recorded, and was easily to use for clinicians. To our knowledge, this study was the first nomogram model for evaluating the risk of LNM in NSCLC based on relevant clinicopathological characteristics, and we also developed a dynamic nomogram application for clinicians and patients to use worldwide. Moreover, other than conventional univariate analysis, LASSO regression was used to select optimal predictive variables, which effectively performed well in reducing the dimension of data (29, 30). Finally, besides the internal validation, the external validation was also used to assess the model's calibration, since there was a large enough number of patients.

Furthermore, the potential limitations of our study should be noted. First, this study cohort only consisted of patients from a single center, which was not representative of patients in other hospitals. A more accurate nomogram should be developed using data from multiple centers in the future. Second, in spite of undergoing systematic LND, a small group of patients received sublobar resection, which might lead to the underestimate of positive N1 rate. We will conduct further research on their difference. Thirdly, our study did not involve all clinicopathological characteristics. In our previous (20) and other studies (31), maximal standardized uptake value (SUV_max_), was proved to be an impressive predictive factors for LNM, but was not included. That is because PET scan was not routinely performed for small-size NSCLC, and the variable with many missing values was not suitable for linear regression. Future studies may consider all clinicopathological characteristics as much as possible for developing a globally-applicable nomogram.

## Conclusions

Based on clinicopathological characteristics, this study firstly developed a cost-effective dynamic nomogram for calculating the precise risk of LNM in small-size NSCLC, and will be helpful for clinicians' decision-making.

## Data Availability Statement

The raw data supporting the conclusions of this article will be made available by the authors, without undue reservation.

## Ethics Statement

The studies involving human participants were reviewed and approved by Ethics Committee of Peking Union Medical College Hospital. Written informed consent to participate in this study was provided by the participants' legal guardian/next of kin.

## Author Contributions

Conceptualization: SL, NL, YW, and CH. Methodology: YW, CH, and ZW. Formal analysis: YW, CH, JL, CH, XL, and YC. Investigation: YW, ZW, and LG. Writing—original draft preparation: YW, CH, and ZW. Writing—review and editing: YW, SL, and NL. Supervision: SL. All authors have contributed significantly and in keeping with the latest guidelines of the International Committee of Medical Journal Editors.

## Conflict of Interest

The authors declare that the research was conducted in the absence of any commercial or financial relationships that could be construed as a potential conflict of interest.

## Supplementary Material

Supplement Figure 1 The surface of the dynamic nomogram application.
