# Supplementary figures and images for: An Externally-Validated Dynamic Nomogram Based on Clinicopathological Characteristics for Evaluating the Risk of Lymph Node Metastasis in Small-Size Non-small Cell Lung Cancer
Source: Front Oncol. 2020 Aug 7;10:1322. doi: 10.3389/fonc.2020.01322 (PMC7426394; doi:10.3389/fonc.2020.01322)

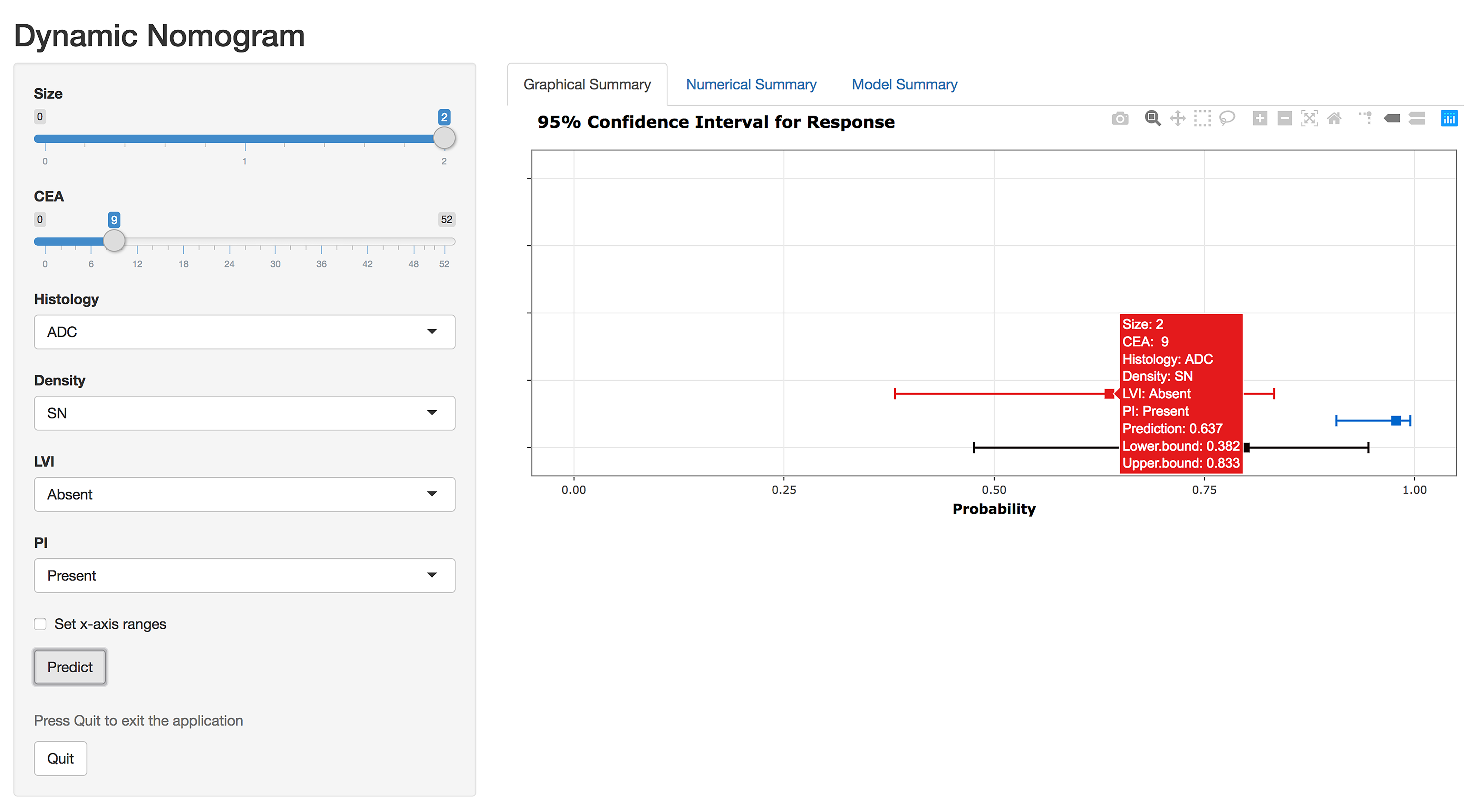

Supplement: Supplement Figure 1 — The surface of the dynamic nomogram application. [file Image_1.TIF]
